# Genome editing of lactic acid bacteria: opportunities for food, feed, pharma and biotech

**DOI:** 10.1093/femsle/fny291

**Published:** 2018-12-18

**Authors:** Rosa A Börner, Vijayalakshmi Kandasamy, Amalie M Axelsen, Alex T Nielsen, Elleke F Bosma

**Affiliations:** The Novo Nordisk Foundation Center for Biosustainability, Technical University of Denmark, Kemitorvet B220, 2800 Kongens Lyngby, Denmark

**Keywords:** genetic tool development, food fermentation, biotherapeutics, phytotherapeutics, synthetic biology, GMO regulation

## Abstract

This mini-review provides a perspective of traditional, emerging and future applications of lactic acid bacteria (LAB) and how genome editing tools can be used to overcome current challenges in all these applications. It also describes available tools and how these can be further developed, and takes current legislation into account. Genome editing tools are necessary for the construction of strains for new applications and products, but can also play a crucial role in traditional ones, such as food and probiotics, as a research tool for gaining mechanistic insights and discovering new properties. Traditionally, recombinant DNA techniques for LAB have strongly focused on being food-grade, but they lack speed and the number of genetically tractable strains is still rather limited. Further tool development will enable rapid construction of multiple mutants or mutant libraries on a genomic level in a wide variety of LAB strains. We also propose an iterative Design–Build–Test–Learn workflow cycle for LAB cell factory development based on systems biology, with ‘cell factory’ expanding beyond its traditional meaning of production strains and making use of genome editing tools to advance LAB understanding, applications and strain development.

## Teaser

Traditional, emerging and future applications of lactic acid bacteria can all benefit from genome editing and a proposed Design–Build–Test–Learn workflow cycle for advancement of strain development.

## INTRODUCTION

**Figure 1.**
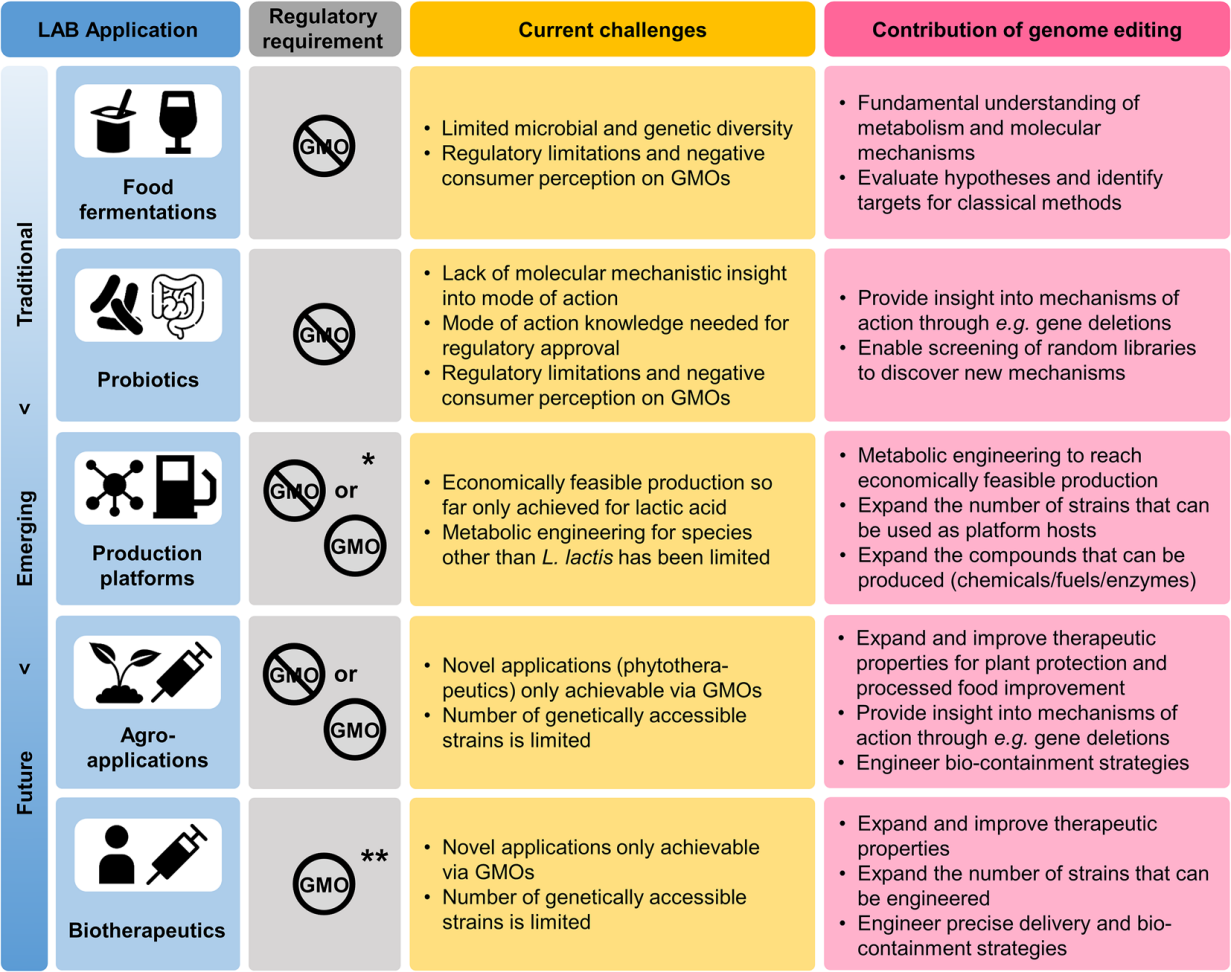
Overview of traditional, emerging and future applications of LAB with the most important contributions of genome editing tools for each, including current regulatory requirements. For all applications, genome editing provides the possibility to make tailored design strains with desired properties, but the direct use of GMO strains is currently limited; here we have depicted only possibilities within the current legislation. *For food ingredients and enzymes: mostly non-GMO via self-cloning. **Currently not approved, but GMOs are needed to reach the desired application.

Lactic acid bacteria (LAB) are a phylogenetically diverse but functionally related group of bacteria comprising the families *Aerococcaceae*, *Carnobacteriaceae*, *Enterococcaceae*, *Lactobacillaceae*, *Leuconostocaceae* and *Streptococcaceae*. They are low-GC, Gram-positive, facultatively anaerobic, non-sporulating bacteria and have a highly fermentative lifestyle, converting a range of sugars into mainly lactic acid. LAB have a long history in different forms of food-related biotechnology and are gaining attention towards novel uses due to their safety for human and animal consumption, metabolic versatility and wide ecological niche adaptation (including industrial-scale fermentations) (Fig. 1).

Genome editing tools for LAB are limited compared to species like *Saccharomyces cerevisiae* and *Escherichia coli*, mostly due to restrictive legislations, and poor consumer acceptance of genetically modified organisms (GMOs) in food. Although LAB were a pioneer group studied for development of genetic tools, with many cloning vectors derived from them still routinely used (De Vos 2011), their tools have mainly focused on being food-grade and less on generating many mutants in a short time. Furthermore, laboratory evolution and random mutagenesis have been widely applied for strain improvement in food applications, as strains resulting from these methods are considered non-GMO. However, such methods do not result in targeted modifications and selection of the right strains is often laborious, despite bioinformatics being highly instrumental to narrow down the initial strain selection (Walsh *et al.*^2017^). The expansion of LAB genome editing tools with a focus on speed to enable fast, clean, targeted and stable genomic modifications for a wide variety of strains is crucial for both fundamental studies and applications.

In this mini-review, we provide a perspective of traditional, emerging and future applications of LAB and how genome editing can advance all these LAB fields, regardless the strain's GMO-status in the final application (Fig. 1). Furthermore, we discuss available tools and suggest how these can be further developed to enable or advance all these applications and fundamental studies, taking also current legislation into account. Finally, we propose an iterative Design–Build–Test–Learn workflow cycle based on systems biology, similar to what is currently used for industrial production platform strains (Palsson 2015; Campbell, Xia and Nielsen 2017). The focus is on engineering single/pure strains and not on microbial community engineering, which has recently been reviewed elsewhere (Sheth *et al.*^2016^; Bober, Beisel and Nair 2018; Zerfaß, Chen and Soyer 2018).

## APPLICATIONS OF LAB AND THE CONTRIBUTION OF GENOME EDITING

### Food fermentations

Fermentation of food and beverages has been carried out for thousands of years (10 000 BC), most likely for food preservation (Prajapati and Nair 2003). The most recent trends in using LAB for food are related to improving properties like nutritional value (e.g. vitamin production), organoleptic quality (e.g. flavour formation) or technofunctionalities (e.g. polysaccharide formation). LAB are also key in primary processing of ingredients such as cocoa and coffee beans (De Vuyst and Weckx 2016; Pereira, Soccol and Soccol 2016) and significantly influence the final product quality (see also *Agro-applications*). With the longest commercial use and an estimated market growth of 7.2% for the next five years (Mordor Intelligence 2018), fermented food is one of the most important economical applications of LAB.

A primary strategy for research in food applications is screening microbial collections (Bourdichon *et al*. 2012). However, with global access to microbial and genetic diversity now limited by the Nagoya Protocol and uncertainties about its interpretation (Darajati *et al.*^2013^; Johansen 2017), achieving genetic variation through genomic manipulation gains relevance. Due to poor consumer acceptance of GMOs, and restrictive legislation, strain development for food applications mainly relies on untargeted and laborious methods based on evolution (Derkx *et al*. 2014; Bachmann *et al*. 2015; Johansen 2018), or on targeted but limited non-GMO methods (Zeidan *et al*. 2017) (see GMO vs non-GMO).

Nevertheless, even without the final GMO-strain ending up in the product, food applications can benefit from genome editing as a research tool (Fig. 1). For example, targeted mutagenesis can be applied to predicted genes for a certain trait to evaluate their function and phenotype (Derkx *et al*. 2014). This is especially important for compounds of which the production is not yet fully understood, such as expolysaccharides (Zeidan *et al.*^2017^). It can also aid in guiding more targeted selection and reduced screening size to select for naturally evolved strains towards the desired modification(s), such as was shown for phage-resistance factor *YjaE* in *Lactococcus lactis* (Stuer-Lauridsen and Janzen 2006). Altogether, improved understanding of compound formation and microbial metabolism will aid in more rational and accelerated efforts to achieve superior properties in food products. Advancing genome editing methods for a wide variety of strains will enable for example screening of mutant libraries, which will further accelerate these processes.

### Probiotics

The World Health Organisation (WHO) has defined probiotics as live organisms that, when administered in adequate amounts, confer a health benefit on the host. Especially *Lactobacillus* species have attracted attention as probiotics, which are used as adjuvant or prophylaxis against many different diseases (Reid 2017; Mays and Nair 2018), as well as in a range of animal husbandries (Syngai *et al*. 2016). The market for probiotics is ever-expanding, with a projected world-wide size of $46.55 billion by 2020 (Salvetti and O’Toole 2017). Nevertheless, the complex molecular mechanistics of modes of action of both probiotics and LAB–host–pathogen interactions are poorly understood (Lebeer *et al*. 2018).

After implementation of EU legislation on health claims in 2009, no probiotics have been granted the right to claim health benefits in the EU. A vast amount of scientific literature indicates beneficial effects of probiotics, but so far in all cases the European Food and Safety Authority (EFSA) considered the scientific substantiation insufficient and rejected all health claims (Dronkers *et al*. 2018). The most important aspects for this are the lack of molecular and mechanistic knowledge of probiotic modes of action**, irreproducibility of trials, as well as strong individual responses of the hosts, and strain-specificity (Glanville *et al.*^2015^; Salvetti and O’Toole 2017; van Pijkeren and Barrangou 2017).

Improving molecular insight into the (dis)functionality of probiotics and observed strain-specificity will be instrumental in achieving the right to health claims and hence further secure markets. Although genomics-, transcriptomics- and metabolomics-based studies are valuable tools (also termed ‘probiogenomics’ in this context) (Guinane, Crispie and Cotter 2016) for identification of potential biomarkers, combining these with genome editing can provide molecular mechanistic insight (Fig. 1) (Lebeer *et al*. 2018). Similar to food, GMOs are not allowed in probiotics, and despite a few examples (Bron *et al*. 2007; Lebeer *et al*. 2018), using GMOs/genome editing as research tool is still relatively underexploited. Advancing genome editing tools to be less time-consuming and more suitable for rapid screening (with suitable fast readout methods) and applicable to a larger number of strains, would potentially enable identification of novel, unpredicted factors. Furthermore, once regulations allow, genome editing could be used to create GMO-/improved probiotics that could for example be combined with biotherapeutics (van Pijkeren and Barrangou 2017).

### Industrial production platforms for green chemicals, fuels and enzymes

A wide range of products can be made through bio-based production via microbial fermentation of biomass-derived sugars to replace fossil resources, such as (building blocks for) plastics, nylons, solvents, fuels, pharmaceuticals and food and cosmetic ingredients. Traditional work horses for this type of cell factories are *E. coli* and *S. cerevisiae*, mostly because their genetic tools are well-developed and their metabolism is relatively well-understood. LAB are gaining interest as alternative hosts for many reasons, which have been extensively reviewed elsewhere (Gaspar *et al.*^2013^; Boguta *et al.*^2014^; Mazzoli *et al*. 2014; Bosma, Forster and Nielsen 2017; Sauer *et al*. 2017; Hatti-Kaul 2018).

One main advantage of LAB is their food-grade safety and adaptation to food-related environments, enabling their use as production platforms in food-related processes. A recent example is the use of metabolically engineered *L. lactis* for ethanol production from lactose in whey, showcasing an alternative of waste valorisation in cheese-making (Liu *et al.*^2016^). Attempts have been made to ferment the whey-lactose with yeasts, but these suffer from low robustness and slow fermentation; using *L. lactis* proved a promising solution on which the company Alcowhey was founded (Liu *et al*. 2016; Jensen *et al.*^2017^). Another LAB-suitable application would be the in-process production of proteins or enzymes for food products by starter or adjunct strains (Matthews *et al.*^2004^). LAB enzymes are also employed for production of food-grade speciality chemicals, pharmaceutical intermediates and nutraceuticals, mostly as whole cell catalysts (Hatti-Kaul 2018). Cofactor regeneration is a challenge in such processes but ingenious solutions using natural substrates have been employed as source of reducing equivalents (Perna *et al.*^2016^).

Except for *L. lactis*, no extensive metabolic engineering has been performed to obtain economically competitive LAB platform organisms (Gaspar *et al.*^2013^; Mazzoli *et al.*^2014^; Bosma, Forster and Nielsen 2017; Sauer *et al.*^2017^). This is largely due to underdeveloped genome editing tools for industrially relevant strains. For example, many *Lactobacillus* and *Pediococcus* spp. have been shown to be more tolerant to several stresses compared to *L. lactis*, but lack widely applicable high-throughput genetic tools (Boguta *et al.*^2014^; Bosma, Forster and Nielsen 2017). Advancing tools for such organisms is important to make use of the wide variety of LAB and their metabolic capacities.

### Agro-applications

To feed the ever-growing world population, crop health is crucial. The use of pesticides is increasingly regarded as undesired, creating the need for organic solutions. Traditionally, research on plant health-promoting microorganisms has focused on Rhizobia, *Bacillus* and *Pseudomonas*; LAB also form a part of the phytomicrobiome of several plant species, but have yet been underexplored (Axel *et al.*^2012^; Lamont *et al.*^2017^). Examples of LAB biocontrol activities are production of reactive oxygen species, bacteriocins (see *Biotherapeutics*), competitive colonisation (overgrowing pathogens) and alteration of the plant immune response (Gajbhiye and Kapadnis 2016; Konappa *et al.*^2016^; Lamont *et al.*^2017^). In many cases, the identity of the antimicrobial compound and which genes encode for it is unknown. Moreover, little is known about the molecular interactions between LAB and plants. Similar to described above for probiotics, genome editing will aid in increasing understanding, which will lead to new possibilities for biocontrol and improvement of plant growth and health (Lamont *et al.*^2017^), expanding LAB to a type of plant probiotics.

Moreover, plant health is related to food and feed for organoleptic and technofunctional properties in the final product. The presence of LAB in the phytomicrobiome has shown to influence for example the processes and tastes of sourdough fermentation of durum wheat flour (Minervini *et al.*^2015^) and milk derived from silage-fed cows (Kalač 2011). A better understanding of the dynamics of the phytomicrobiome in raw material and food processing could guide new applications or technofunctionalities in the food industry.

Altogether, the agro-industry is a promising LAB application field and whereas the use of GMOs in organic farming is currently out of the question, genome editing can be beneficial as a research tool (Fig. 1).

### Biotherapeutics

One of the most promising novel applications of LAB is their medical use in therapeutics, prevention and diagnosis (Mays and Nair 2018). Especially their use as delivery agents of drugs and vaccines is gaining attention. LAB are particularly suitable as they are already generally recognised health-improving agents and safe for human consumption. Efforts using LAB as biotherapeutics have mostly focused on gastrointestinal tract-related ailments using the strains as oral vectors, leveraging their capacity to survive stomach acids and adhere to the intestinal epithelium (De Moreno De Leblanc *et al.*^2015^; Hwang *et al.*^2016^; Carvalho *et al.*^2017^; Durrer, Allen and Hunt von Herbing 2017). LAB are also being developed for mucosal (vagina and mouth) delivery of molecules and as vaccines (Wang *et al.*^2016^), as well as for wound treatment (Vågesjö *et al.*^2018^). Many LAB naturally produce antimicrobial peptides (e.g. bacteriocins), which are currently commercialised in the purified form for veterinary use (Ahmad *et al.*^2017^). These compounds have demonstrated high specificity and potency *in vivo*; they are a potential alternative to fight the rising antimicrobial resistance, and also have applications in food preservation and probiotics (Yang *et al.*^2014^; Mathur *et al*. 2017). Targeted delivery via synthetic biology can potentiate their use as antimicrobial agents of the future. Also, CRISPR-based antimicrobials hold great promise (Pursey *et al.*^2018^) and would be highly interesting to develop also using LAB. Another attractive field is the use of LAB for diagnosis by acting as biosensors inside or outside of the body (Lubkowicz *et al.*^2018^).

The microbial therapeutics and diagnostics market is estimated to occupy close to 79% of the therapeutics segment by 2030 with annual growths over 80% from 2019 onwards, attracting boosts in funding and investment (Microbiome Therapeutics and Diagnostics Market (2nd Edition), 2017–2030 2017). As a new field, there are no commercially available LAB-biotherapeutics yet, besides the non-GMO ones composing the community in human faecal transplantations approved by the FDA (FDA 2016). This is expected to change soon, as the first clinical trials by pharmaceutical companies with live-engineered biotherapeutics are on-going (Bron and Kleerebezem 2018). Although more research is required regarding efficiency, fundamental questions and safety, LAB as biotherapeutics can bring a revolution in personalised and precise medicine (Mays and Nair 2018).

Stable and tuneable modifications via genome editing and synthetic biology are crucial in this field for the addition of the therapeutic compounds to the microbial delivery host, as well as for the insertion of regulation mechanisms, delivery strategies and biocontainment systems (Mays and Nair 2018) (Fig. 1). The absence of genetic markers, such as antibiotics, in the final strain is essential to avoid risk of transferring antibiotic resistance to pathogens inhabiting the host. Furthermore, the current tools are mostly limited to a few strains (*L. lactis*) while several *Lactobacillus* spp. have proven a more promising target group due to prolonged survival and colonisation of the gastrointestinal tract. Currently, their limited genetic accessibility and toolbox restrain their use (Allain *et al.*^2015^; van Pijkeren and Barrangou 2017; Bron and Kleerebezem 2018). Finally, as with probiotics, better understanding of the interactions with the host on a molecular and cellular level is needed to enable full development of LAB as biotherapeutics (Fig. 1) (van Pijkeren and Barrangou 2017).

## OVERVIEW OF LAB GENOME EDITING TOOLS: CURRENT AND FUTURE

**Figure 2.**
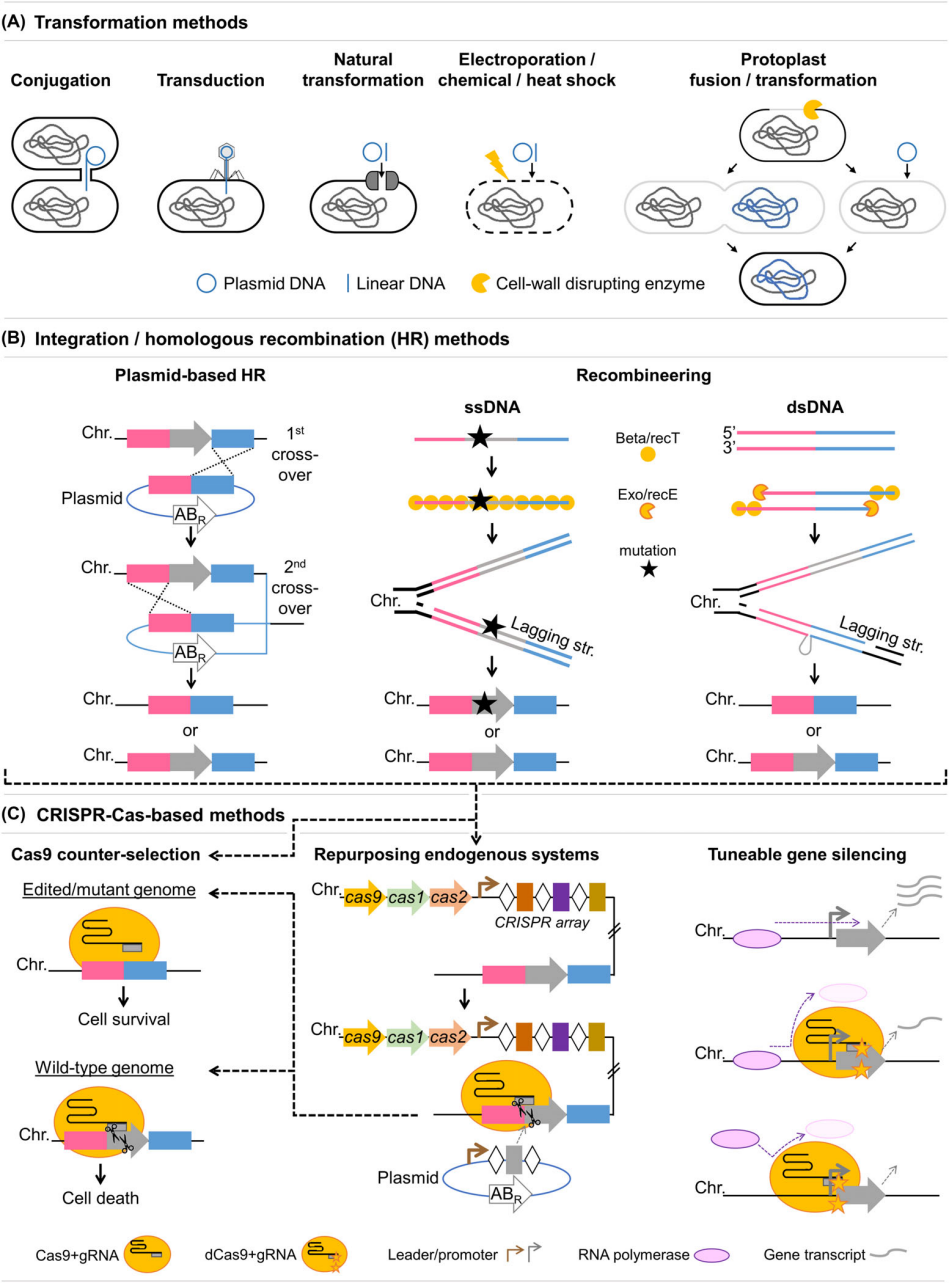
Schematic overview of transformation and genome editing methods currently available for LAB. Only methods that result in clean mutations (or silencing) and that can be targeted to any desired site in the genome are shown. The grey arrow on the chromosomes represents the target gene of interest. Abbreviations: Chr.: chromosome; str.: strand; AB_R_: antibiotic resistance; ssDNA: single stranded DNA; dsDNA: double stranded DNA; gRNA: guide RNA, which can be either a single guide (sgRNA) or a dual crRNA:tracrRNA. **(A)**, Transformation methods. For electroporation/chemical/heat shock transformation, the yellow flash indicates any of these external treatments (electrical pulse, chemical treatment or heat shock). For the protoplast-based method, the left arrow indicates protoplast fusion of two different cells and the right arrow indicates transformation of protoplasts. **(B)**, Integration/homologous recombination (HR) methods. *Plasmid-based HR* uses the native recombination machinery. dsDNA *recombineering* requires the expression of a phage λ- or Rac prophage-derived exonuclease (Exo or RecE) and an ssDNA binding protein (Beta or RecT), whereas ssDNA recombineering only requires the single-stranded binding protein. In the case of the λ-Red system, also Gam can be added, which inhibits host DNA exonucleases (Van Pijkeren and Britton 2012; Pines *et al*. 2015). A marker can be introduced within the homologous regions but this does not result in clean mutations. Without marker insertion (as depicted here), the result can be either wild-type or mutant, which need to be verified by PCR, and for which Cas9 can be used as *counter-selection* as depicted in C. **(C)**, CRISPR-Cas-based editing and silencing tools. The two methods on the left could be used in combination with any of the integration methods shown in B. For endogenous systems, a type II system is depicted here with Cas9 as effector molecule, but also other endogenous systems could be used for both editing and silencing, although this has not yet been shown in LAB (Luo *et al.*^2015^; Rath *et al*. 2015; Li *et al*. 2016). *Repurposing endogenous systems* to target the organism's own genome can be achieved by plasmid-based expression of the native minimal CRISPR array (leader and two repeats), or a synthetic single guide RNA based on the native system, together with desired spacer(s) to target a (or multiple) gene(s) of interest. Prerequisites are that the native system is active under the *in vivo* editing conditions and that the different components and the PAM recognised by the system are characterised (Crawley *et al.*^2018^). *Gene silencing* using catalytically inactive Cas9 (‘dead’ Cas9, dCas) has only been shown as proof of principle in *L. lactis* (Berlec *et al.*^2018^) but the tuneable nature has not yet been exploited in LAB, but several methods for this are available and have been shown in other organisms (Mougiakos *et al.*^2016^).

Several methods have been developed for making genomic modifications in LAB, including food-grade ones that result in strains labelled as non-GMO (see also GMO vs non-GMO). These are still very useful for the many LAB applications where GMOs are not allowed, and continue to gain interest (Bron *et al.*^2019^). However, to further expand LAB applications as described above, the following advancements are required: (i) increase editing speed, (ii) methods for multiplexing (i.e. simultaneous modification of several genomic loci in one editing round) and (iii) broaden the range of strains that can be transformed and edited. This section discusses how these can be achieved via existing methods and future developments, following the different steps of the editing process from transformation to mutant construction (Fig. 2). We focus on methods that can be targeted to any desired place in the genome with stable and marker-free results. Also, screening/readout systems for the generated mutants are required, but as this is a field in itself and out of the scope of this review, the reader is referred to other recent publications (Chen *et al.*^2017^; Duarte, Barbier and Schaerli 2017; Emanuel, Moffitt and Zhuang 2017; Longwell, Labanieh and Cochran 2017).

### Transformation (DNA transfer) and genetic accessibility

Transformation (the process to introduce DNA) is the critical first step towards any genome editing and can be achieved via naturally occurring or artificial methods (Fig. 2A). Natural methods, particularly conjugation, have been exploited to achieve non-GMO LAB strains (Pedersen *et al.*^2005^; Derkx *et al.*^2014^; Bron *et al.*^2019^). Conjugative plasmids and transposons are very common in LAB, but the details of conjugative mechanisms are not fully understood and this field needs improvement to widen its applicability (Kullen and Klaenhammer 2000; Dahmane *et al.*^2017^; Bron *et al.*^2019^). Phage transduction is a wide-spread phenomenon in LAB but not yet frequently harnessed for targeted DNA exchange (Bron *et al.*^2019^). It also is a potential tool for human microbiome engineering (Sheth *et al.*^2016^). Natural competence, in which exogenous DNA translocates through a native DNA uptake machinery, is well-known in *Streptococcus* (Gardan *et al.*^2009^; Muschiol *et al.*^2015^), but only recently identified and achieved in *Lactococcus* (David *et al.*^2017^; Mulder *et al.*^2017^). The abundance of natural competence is likely underestimated (Blokesch 2016; Bron *et al.*^2019^) and the new findings might pave the way for natural transformation in other LAB that are so far considered non-genetically accessible.

In artificial methods, cells need to be made competent through for example washing with cell envelope-weakening solutions, after which external agents are used for cell permeabilisation and transformation. Electroporation is the most suitable method for high-throughput purposes due to its simplicity, efficiency and wide applicability (Landete 2017). Generalised electroporation protocols have been successfully used to transform a wide range of LAB strains. Although these studies indicate that the majority of LAB is genetically accessible through electroporation, efficiencies varied strongly among strains and protocols need to be optimised (Landete *et al.*^2014^; Bosma, Forster and Nielsen 2017). A method with low efficiencies and less suitable for targeted modification but suitable for the large-scale exchange of genomic DNA for e.g. evolutionary engineering via genome shuffling, is protoplast fusion (Mercenier and Chassy 1988; Patnaik *et al.*^2002^).

Bacteria, including LAB, have evolved defence strategies against foreign DNA, such as restriction modification (RM) and CRISPR-Cas systems or combinations thereof (Dupuis *et al*. 2013). In RM-systems, a set of enzymes discriminates self from non-self DNA by methylating it and cleaving the invading DNA (Vasu and Nagaraja 2013). Recent reports have shown the existence of ‘phase-variable’ RM-systems in LAB, which result in variable methylation patterns (De Ste Croix *et al.*^2017^), and as of yet ununderstood restriction-like factors that mutate during the editing process (Ortiz-Velez *et al.*^2018^). Limitations for introducing and maintaining foreign DNA have been mainly related to RM-systems and to further develop any genome editing method, it is often required to bypass these (Teresa Alegre, Carmen Rodríguez and Mesas 2004; Spath, Heinl and Grabherr 2012; Joergensen *et al.*^2013^).

### Genome editing (DNA integration)

Detailed descriptions of traditional and currently available LAB genome editing methods are provided in several recent reviews (Bosma, Forster and Nielsen 2017; Landete 2017; Hatti-Kaul 2018). Here, we outline the main steps and bottlenecks in LAB genome editing and focus on how recent advancements can be further developed to improve this. Classically, LAB genome editing for targeted genomic modifications is based on integrative plasmids to insert or remove a gene of interest via two crossover events using the cells’ native recombination machinery (Fig. 2B). Steps in this procedure that can be time-consuming are the selection of integrants (i.e. cells that have correctly integrated the exogenous DNA over the homologous regions) and the curing of the integrative plasmid after homologous recombination (HR). Several tools have traditionally been used to make these processes more efficient, such as thermo-sensitive and suicide vectors and counter-selectable markers. Instead, the more recently developed method of recombineering enables direct integration of linear ssDNA or dsDNA oligos into the genome with the help of phage-derived recombination systems (Fig. 2B). This avoids curing integrative plasmids from the cells and cloning of HR regions, making this method more suitable for high-throughput purposes. However, recombineering requires identification of phage-derived proteins and optimisation of the system for each new strain, and hence has been developed for less strains than plasmid-based systems. Recombineering has been established in *Lactococcus lactis*, *Lactobacillus reuteri*, *Lactobacillus gasseri* (Van Pijkeren and Britton 2012), *Lactobacillus casei* (Xin *et al.*^2018^) and *Lactobacillus plantarum* (Yang, Wang and Qi 2015; Leenay *et al.*^2018^). Also, site-specific recombination systems based on phage integrases and phage attachment sites have been developed for LAB, often as food-grade systems (Alvarez, Herrero and Suárez 1998; Brøndsted and Hammer 1999; Grath, van Sinderen and Fitzgerald 2002). Although these systems are very valuable and applicable to a wide range of strains for stable integrations, we will not go into detail here as they are limited to integrations into specific locations in the genome (i.e. in the phage attachment sites only).

For both plasmid-based and recombineering methods, a critical bottleneck step is the selection of correctly edited mutants. Plasmid-based editing can result in either mutants or wild-type revertants (Fig. 2B), and recombineering efficiencies are inherently low, resulting in large amounts of wild-type cells: for ssDNA recombineering in *L. reuteri*, efficiency was 0.4%–19% (Pijkeren and Britton 2014). This creates an often laborious and time-consuming PCR-based screening process. Marker insertion-and-removal systems such as Cre-*lox* have been employed in some LAB to overcome this, but such methods leave small scars and hence are not fully clean (Yang, Wang and Qi 2015; Xin *et al*. 2018). To increase efficiencies of clean editing systems, it is necessary to establish selection tools for mutants, or counter-selection tools against wild-types. Most recently, CRISPR-Cas9-technology has proven a powerful counter-selection tool in bacteria (Fig. 2C) and to significantly speed up and advance engineering (Mougiakos *et al.*^2016^, ^2018^).

### CRISPR-Cas-based genome editing

CRISPR-Cas systems in nature function as prokaryotic adaptive immune systems (Barrangou *et al.*^2007^; Brouns *et al.*^2008^) and although a wide variety exists (Koonin, Makarova and Zhang 2017), Cas9—the endonuclease of Type II CRISPR-Cas systems—has gained most fame as a versatile genome editing tool. When directed to its target DNA by a provided guide RNA and recognising its target next to a short DNA motif called protospacer adjacent motif (PAM), Cas9 creates blunt dsDNA breaks (Fig. 2C). Whereas eukaryotes can repair such breaks by non-homologous end joining (NHEJ), this system is absent or inactive in most bacteria (Bowater and Doherty 2006). Hence, they are unable to repair Cas9-induced breaks, which creates a powerful counter-selection tool against wild-type cells as these will be killed due to Cas9 cleavage (Fig. 2C) (Mougiakos *et al*. 2016). In *L. reuteri*, Cas9-based selection of mutants after ssDNA recombineering increased the efficiency from 0.4%–19% to 100% (Oh and Van Pijkeren 2014). Cas9-based editing has now been established in *L. reuteri* together with ssDNA recombineering (Oh and Van Pijkeren 2014), in *L. plantarum* with dsDNA recombineering and plasmid-based HR (Leenay *et al*. 2018), and with plasmid-based HR in *L. lactis* (van der Els *et al.*^2018^). Cas9 has also been used for removal of large mobile genetic elements in *Streptococcus thermophilus* (Selle, Klaenhammer and Barrangou 2015) and *L. lactis* (van der Els *et al.*^2018^).

A major challenge of using Cas9 in bacteria is that its activity must be tightly controlled to allow HR-based genome editing before killing wild-type cells, requiring tightly controllable expression systems or multiple plasmids and transformation rounds. A nickase-variant of Cas9 makes single stranded nicks instead of double stranded breaks due to a mutation in one of the two active sites of Cas9. These nicks are less lethal, and are furthermore suggested to enhance HR (Song *et al*. 2017). The nickase was used together with an integrative plasmid in *L. casei* with an efficiency up to 65%, requiring only a single transformation round (Song *et al.*^2017^).

Establishing HR/Cas9-based editing methods is not trivial due to strong and yet ununderstood strain-specific differences. A direct comparison of recombineering- and plasmid-based methods in *L. plantarum* showed several strain-specific differences in efficiencies (Leenay *et al*. 2018). Moreover, Cas9 has shown to be toxic in certain bacteria, for which subsequently alternative CRISPR-Cas systems such as Cas12a (formerly Cpf1) have been successful (Jiang *et al.*^2017^). Several alternative Cas9s and other CRISPR-Cas-systems are now being characterised for genome editing in other microorganisms, showing advantages such as wider applicability, specificity, stability or less toxicity (Jiang *et al*. 2017; Mougiakos *et al*. 2017; Nakade, Yamamoto and Sakuma 2017). Evaluating such alternative systems in LAB might open new possibilities for CRISPR-Cas-based editing in a wider range of LAB. Furthermore, the repurposing of endogenous CRISPR-Cas systems, which are abundantly present in LAB (Sun *et al*. 2015), into counter-selection systems is a promising recent approach for broadening the number of engineerable species (Fig. 2C) (Crawley *et al.*^2018^).

All reported genome modifications in LAB so far only make one modification at a time, while multiplexing would be crucial for many applications including fundamental studies. Multiplexing is complicated with plasmid-based HR and would strongly benefit from establishing recombineering methods for more strains. Another interesting option in this regard is the recently developed base editing, in which a catalytically impaired Cas9-variant is coupled to a cytidine deaminase that does not make DNA breaks, but C to T (or G to A) substitutions (Kim *et al.*^2017^). This can be used to make targeted point mutations to create premature stop codons and inactivate genes without the need for HR. It has only been used in few bacteria (Kim *et al.*^2017^; Eid, Alshareef and Mahfouz 2018; Zheng *et al.*^2018^) and not yet for LAB.

### Gene silencing and synthetic biology

A catalytically ‘dead’ Cas9-variant (dCas9) can be used for high-throughput and tuneable gene silencing instead of gene editing: mutating both Cas9-active sites creates a catalytically inactive Cas9 that binds DNA but does not cleave it (Bikard *et al.*^2013^; Qi *et al.*^2013^). This has not been exploited for LAB other than as proof of principle in *L. lactis* (Berlec *et al.*^2018^) and would be a highly valuable addition to the toolbox. No HR is needed, creating an easy screening tool with high potential for multiplexing. Although not yet used for this purpose in LAB, its tuneable nature creates a powerful tool for investigating downregulation of essential genes (Fig. 2C) (Peters *et al.*^2016^; Mougiakos *et al.*^2018^; Rousset *et al.*^2018^).

Regarding synthetic biology developments, improving regulatory control systems is highly desirable, especially for bio-therapeutic applications. Particularly, promoters that can be induced in e.g. the gut by the host metabolites to control gene expression *in vivo* at the targeted location (Bober, Beisel and Nair 2018), as well as bio-containment strategies, which are crucial for safety (Wegmann *et al.*^2017^). Systems based on quorum-sensing or reciprocal transcriptional repression systems have been used for inducing autolysis in *E. coli* (Chan *et al.*^2016^; Hwang *et al.*^2017^) and could be adapted to LAB. Gene circuits construction is also important for the development of bacterial biosensors, where engineered strains can detect certain molecules related to a disease in the human host.

### GMO vs non-GMO

Regulations surrounding GMOs are complex and consumer acceptance plays an important role in the reluctance to use GMOs, especially in food. In the EU, GMOs are not allowed in the final product (i.e. as food, probiotics or bio- and phytotherapeutics), but are allowed as contained production hosts (i.e. as producers of chemicals, fuels and enzymes in which the organism remains within a factory/reactor) (Johansen 2018). Even if the microorganism does not end up in the final product but is used to produce food ingredients (e.g. enzymes), lack of consumer acceptance of GMO-products puts pressure on food and also ingredient companies to use GMO-free enzymes (Derkx *et al.*^2014^). Therefore, even contained microorganisms in such cases should be non-GMO.

For these reasons, genome editing tools for LAB traditionally focus on systems labelled as non-GMO. Next to strains created via random mutagenesis or laboratory evolution, the current EU legislation considers strains generated by natural gene transfer methods (e.g. conjugation; transduction) as non-GMO, provided none of the involved strains is a GMO (Sybesma *et al.*^2006^; Johansen 2017). For contained use, microorganisms are also considered non-GMO if they are made by ‘self-cloning,’ which means modification of a strain with DNA taken from the strain itself or from a very close relative. This may involve recombinant vectors as long as these consist of DNA from this same or closely related strain (Meacher 2000; Verstrepen, Chambers and Pretorius 2006; Landete 2017). By definition, this also means that clean deletion mutants created with such LAB-vectors are considered non-GMO (De Vos 1999). Self-cloning and its ‘non-GMO’ label is only allowed for contained use and the organisms created by such methods are not allowed in the final product (Sybesma *et al.*^2006^; Johansen 2018), or should be inactivated at the end of the process.

Regarding advanced genome editing tools (e.g. recombineering; CRISPR-Cas), if the tool vectors come from species related to the target strain, they could be considered as ‘self-cloning,’ having the added advantage of being clean/marker-free if using appropriate methods (Fig. 2). Targeted genomic modifications would result in a similar genotype as the wild-type strain, plus or minus a specific gene that could also have been edited by a classical method like random mutagenesis (Johansen 2017). It has been argued by several players in the field that it is questionable whether a strain obtained via random mutagenesis (currently allowed for human consumption) is safer than if that same strain was obtained via targeted and clean self-cloning methods (Johansen 2017; Bron *et al.*^2019^). However, the EU-court has recently ruled against allowing such new genome editing methods (including CRISPR-Cas) as ‘non-GMO,’ whereas in the USA Cas9-edited plants have recently been allowed (Callaway 2018; Court of Justice of the European Union 2018). This does not change the current situation, but it does mean that allowance of any form of non-contained GMOs, including via clean methods, is unlikely in the near future in the EU. Nevertheless, information dissemination for public awareness and further investigation of potential long-term effects of GMOs is still needed (Sybesma *et al.*^2006^; Fears and Ter Meulen 2017; Johansen 2017; Csutak and Sarbu 2018).

## CONCLUSIONS AND OUTLOOK

In the long term, genome editing could be used to create tailored LAB strains for properties on demand for any given application. This is currently done for e.g. production platforms. For more traditional applications related to human consumption, this possibility is restrained by regulations and consumer opinion. Nevertheless, genome editing can be applied for strain advancement in an indirect way as a research tool, by improving knowledge on the strain itself and the relations with its hosts, as well as provide guidance towards targets for modifications using ‘natural’ or accepted editing methods avoiding a GMO label. To enable such developments, more advanced genome editing tools need to be developed, for a wider range of LAB. This includes making more strains genetically accessible for transformation and establishing recombineering and CRISPR-Cas-based methods, including multiplex genome editing and silencing.

**Figure 3.**
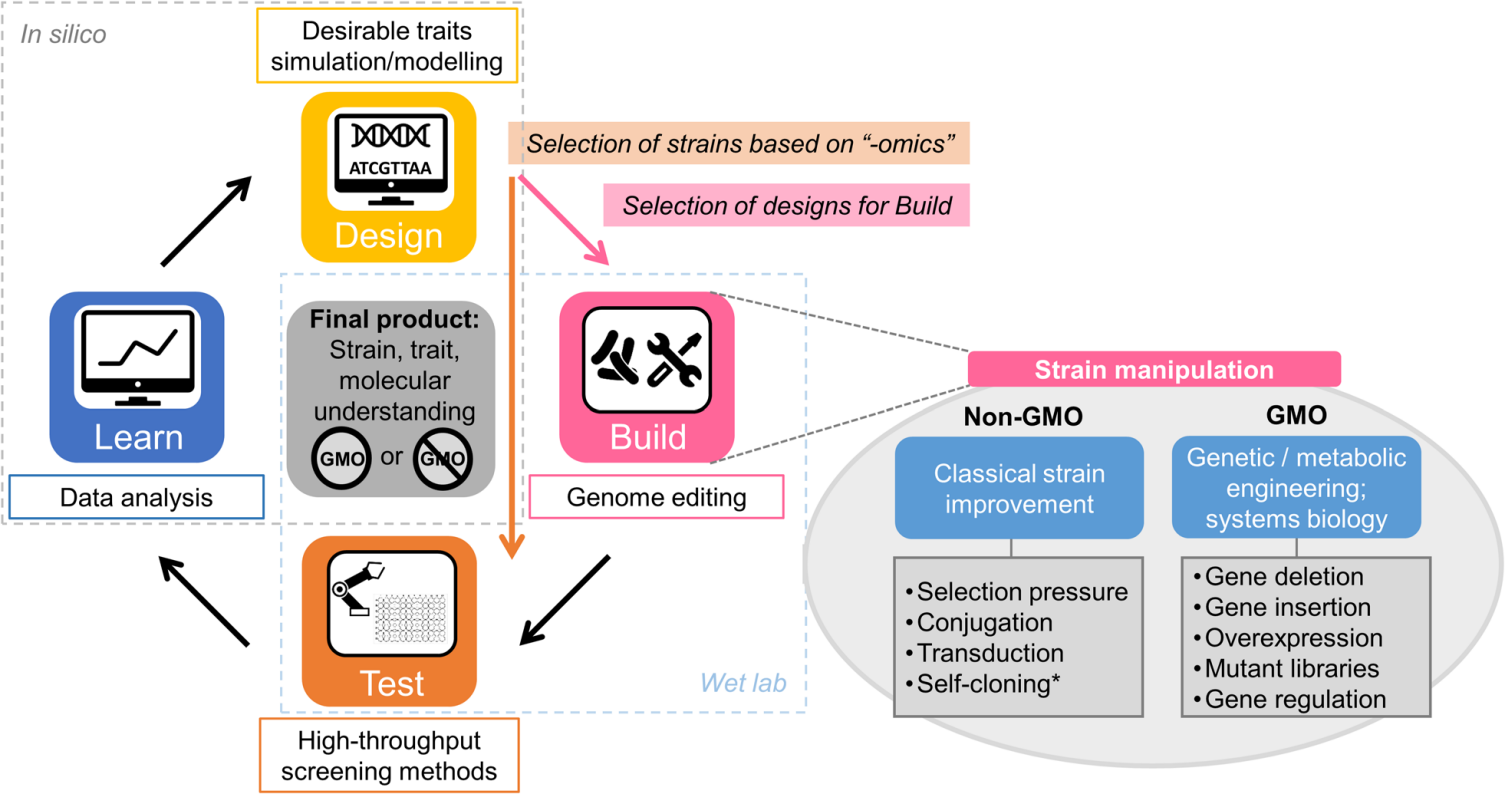
Iterative Design–Build–Test–Learn workflow for cell factory development. Proposed workflow generally applicable to all forms of cell factories discussed in this review based on systems biology for rational and advanced strain development. Adapted for LAB from the ‘classical’ industrial workflow described elsewhere (Palsson 2015; Campbell, Xia and Nielsen 2017). In a full cycle, strains that pass through *Build* are manipulated by genome editing methods that result in GMO or non-GMO strains (see GMO vs non-GMO). For targeted engineering, the desired genotypes are planned in the *Design* step. The same workflow can be applied to a collection of strains where no genetic modification is performed, but rather goes directly to experimental screening (*Test*). In this case, *in silico* work can aid in the pre-selection of the strains to be tested experimentally based on genomic information (*Design*). This can also be a second cycle after a first one which included genome editing to determine targets. In all cases, experimental data analysis and computer integration on e.g. genome scale models (*Learn*) will bring information that can be used for planning and designing the next iterative cycle. *In the EU, self-cloning is allowed for contained use, but not for non-contained applications such as food and probiotics.

For all applications described here, whether the final strain is a GMO or not, the LAB can be considered as microbial cell factories, and an iterative Design–Build–Test–Learn workflow could be applied similar to that used in the development of traditional industrial biotechnology strains for green chemical production (e.g. *E. coli; S. cerevisiae*) (Palsson 2015) (Fig. 3). Such a systems biology-based workflow has been shown to significantly speed up the process of cell factory development by combining genome editing and synthetic biology, *in silico* prediction and models, and high-throughput methods/automation (Campbell, Xia and Nielsen 2017). To be applied to the wide variety of LAB applications described here, this workflow could be used as in a classical metabolic engineering approach, generating GMO or non-GMO strains depending on the modification method used, but also as a research tool for fundamental understanding of the strains by designing mechanistically targeted experiments with non-GMOs as final result (Figs 1 and 3). Accelerated methods for strain construction, selection and screening/readout tools are crucial for advancing this strategy. Also, expanding and improving genome-scale metabolic models is needed to strengthen the *in silico* part (Stefanovic, Fitzgerald and McAuliffe 2017; Rau and Zeidan 2018). An ever-increasing interest in LAB and the advances in genome editing and biotechnological developments will undoubtedly provide breakthrough solutions for innovation in the wide and ever-expanding applications of LAB.
